# Surface Structure Characterization of Shape and Size Controlled Pd Nanoparticles by Cu UPD: A Quantitative Approach

**DOI:** 10.3389/fchem.2019.00527

**Published:** 2019-07-31

**Authors:** Emmanuel Garnier, Francisco J. Vidal-Iglesias, Juan M. Feliu, José Solla-Gullón

**Affiliations:** Instituto de Electroquímica, Universidad de Alicante, Alicante, Spain

**Keywords:** nanoparticles, shape, size, surface structure, electrocatalysis, Cu UPD

## Abstract

The search for new surface sensitive probes that characterize the surface structure of shape and size-controlled nanoparticles is an interesting topic to properly understand the correlations between electrocatalytic properties and surface structure at the nanoscale. Herein, we report the use of Cu UPD to characterize, not only qualitatively but also quantitatively, the surface structure of different Pd nanoparticles with controlled particle shape and size. Thus, Pd nanoparticles with cubic, octahedral and rhombic dodecahedral shapes, that is, with preferential {100}, {111}, and {110} surface structures, respectively, were prepared. In addition, cubic Pd nanoparticles with different particles sizes and spherical (2–3 nm) Pd nanoparticles were also synthesized. Based on the Cu UPD results on Pd single crystals, a new approach is proposed to qualitatively and quantitatively determine the percentages of {100}, {111}, and {110} surface domains present at the surface of the different shape and size controlled Pd nanoparticles. The results reported clearly show the benefits of this Cu UPD to get detailed information of the surface structure of the nanoparticles according to their particle shape and size.

## Introduction

The surface structure of a metal plays a key role on its electrocatalytic properties. This is well-established thanks to the extensive and intensive studies performed with metal single crystal electrodes for most of the electrocatalytic reactions of interest (Korzeniewski et al., 2012). In addition, this surface structure sensitivity has been also reported to be of outstanding relevance for nanoparticulated metal materials, particularly from the availability of shaped metal nanoparticles and their application in Electrocatalysis (Solla-Gullón et al., 2006, 2008b; Solla-Gullon et al., 2011; Tian et al., 2007; Erikson et al., 2011, 2016a; Shao et al., 2011b; Jin et al., 2012; Vidal-Iglesias et al., 2012; Figueiredo et al., 2013). Accordingly, it is widely accepted that by controlling the shape of the nanoparticles, nanoparticles with a well-defined surface structure, that is, well-defined surface atomic arrangement and coordination, can be obtained (Tian et al., 2008; Zhou et al., 2009). Similarly, as reported in previous literature (Kinoshita, 1990; Shao et al., 2011a), when changing the particle size the surface structure of these sized-controlled metal nanoparticles is also strongly affected. In this way, it is highly important to develop sensitive probes to characterize the surface structure of these materials, in order to subsequently establish correct relationships between the surface structure and electrocatalytic reactivity at the nanoscale.

Among others, the use of pure electrochemical probes has been shown to be a powerful technique to successfully characterize the surface structure of different types of shape-controlled metal nanoparticles (Solla-Gullón et al., 2004, 2008a; Chen et al., 2012; Farias and Feliu, 2019; García-Cruz et al., 2019; Mayet et al., 2019). In addition, it is worth noting that this electrochemical surface structure characterization is (i) statistically representative, because thousands of nanoparticles are measured simultaneously, and (ii) performed in a similar environment than those that will be employed during the electrochemical reactions. Thus, for instance, in the case of platinum, the so-called hydrogen and anion adsorption/desorption region as well as the adsorption of bismuth and germanium adatoms have clearly shown the benefits of the electrochemical approach to gain relevant and very detailed information about the surface structure of the different shaped Pt nanoparticles (Solla-Gullón et al., 2004, 2008a; Rodríguez et al., 2005). Similarly, for gold surfaces, the underpotential deposition (UPD) of Pb has been shown to be an excellent probe for obtaining an estimation of the different surface domains present on various types of Au nanoparticles (Hernández et al., 2009; Chen et al., 2017; Jeyabharathi et al., 2018; García-Cruz et al., 2019). However, in the case of palladium, and despite some preliminary attempts (Jin et al., 2012; Solla-Gullon et al., 2015; Higuchi et al., 2018), a systematic analysis to qualitatively and quantitatively determine the presence of well-defined surface domains at the surface of different shape and size controlled Pd nanoparticles is still missing. This analysis will be based on the Cu UPD process on Pd which was reported to be highly sensitive to the surface structure as previously illustrated with Pd single crystal surfaces (Chierchie and Mayer, 1988; Cuesta et al., 1999; Vidal-Iglesias et al., 2006, 2007; Korzeniewski et al., 2012; Mayet et al., 2019).

A detailed characterization of the surface structure of the shape and size of controlled Pd nanoparticles will importantly help to provide better comprehension of the interesting electrocatalytic properties of these Pd nanomaterials toward relevant electrochemical reactions. This includes the oxygen reduction (Erikson et al., 2011, 2016a,b; Shao et al., 2011b, 2013a; Zhang et al., 2013), oxidation of some small organic molecules (Zhang et al., 2011; Jin et al., 2012; Vidal-Iglesias et al., 2012; Arjona et al., 2013; Kannan et al., 2013; Shao et al., 2013a; Chen et al., 2014; Yu et al., 2019), and CO_2_ reduction (Klinkova et al., 2016; Huang et al., 2017; Gao et al., 2018) among others, because the surface structure of the nanoparticles, rather than the shape or size, is the key point determining the resulting electrocatalytic activity (obviously, the atomic composition is also extremely relevant). Consequently, the aim of this manuscript is to develop an electrochemical approach, based on the Cu UPD reaction of Pd, to quantify the percentage of {100}, {110}, and {111} surface domains on different shape and size-controlled Pt nanoparticles.

## Materials and Methods

### Synthesis of Nanoparticles

The different Pd nanoparticles used in the present manuscript were prepared using some previously described methodologies. Shape-controlled, including cubic (Pd_Cub_), octahedral (Pd_Oct_), and rhombic dodecahedral (Pd_RD_) Pd nanoparticles, ideally enclosed by {100}, {111}, and {110} facets, respectively, were obtained using a seed-mediated method (Niu et al., 2010). In brief, this methodology is based on the growth of Pd nanocubes (~20 nm) which act as seeds, in the presence of L-ascorbic acid (AA), H_2_PdCl_4_, cetyltrimethylammonium bromide (CTAB), and NaI as a reducing agent, Pd precursor, capping agent, and additive, respectively, at a specific reaction temperature during a particular reaction time. By controlling some of these experimental parameters, different shapes can be obtained. Table 1 summarizes the experiment parameters used for each case. In a typical synthesis, the required amount of NaI solution was added to 25 mL of a 100 mM CTAB solution at a given temperature (in an oil bath). Then, 625 μL of a 10 mM H_2_PdCl_4_ solution and 200 μL of the ~20 nm as-synthesized seed Pd nanocube solution were added. Finally, a given volume of a freshly prepared 100 mM ascorbic acid solution was added and thoroughly mixed. This final solution was kept at the designated temperature during the required reaction time. The reaction was stopped by centrifugation (6,000 rpm, 10 min) and samples were twice centrifuged and dispersed in water. As described in previous contributions, an additional alkaline cleaning step is needed (Erikson et al., 2011; Montiel et al., 2017). A NaOH pellet was added to the water solution containing the nanoparticles. The incorporation of the NaOH produces the destabilization of the nanoparticles which precipitate. After complete precipitation, the nanoparticles were washed 3–4 times with ultrapure water (Milli-Q) and finally stored in a water suspension.

**Table 1:** Summary of the experimental conditions for the growth of cubic (Pd_Cub_), octahedral (Pd_Oct_), and rhombic dodecahedral (Pd_RD_) Pd nanoparticles at a controlled temperature (T) during *t* hours.

	**T/K t/h**	**NaI**	**CTAB**	**H_**2**_PdCl_**4**_**	**Seeds**	**Ascorbic acid**
Pd_Cub_	328 K 4.5 h	25 μL 0.1 mM	25 mL 100 mM	625 μL 10 mM	200 μL	100 mM 250 μL
Pd_Oct_	313 K 15 h	125 μL 1 mM	25 mL 100 mM	625 μL 10 mM	200 μL	100 mM 250 μL
Pd_RD_	353 K 1 h	125 μL 1 mM	25 mL 100 mM	625 μL 10 mM	200 μL	100 mM 500 μL

To evaluate the effect of the particle size, and based on previous literature (Niu et al., 2008; Lim et al., 2010; Erikson et al., 2016a), different size-controlled Pd nanocubes (~20 and ~10 nm) were also prepared. In both cases, the synthesis is based on a one-step approach. Very briefly, for the preparation of ~20 nm Pd nanocubes (these Pd nanocubes are the ones used as seeds for the seed-mediated method described above, Pd^20^_Cub_), 10 mL of 10 mM H_2_PdCl_4_ and 200 mL of 12.5 mM CTAB were mixed together and placed in an oil bath at 95 °C for 5 min. Then, 1.6 mL of a 100 mM ascorbic acid solution was incorporated and the reaction was allowed to proceed for 30 min at 95°C. After the solution had cooled down, the solution was twice centrifuged and dispersed in water. Finally, a NaOH pellet was added to this solution. Once the nanoparticles were completely precipitate, they were washed 3–4 times with ultrapure water and finally stored in a water suspension. For the synthesis of the ~10 nm Pd nanocubes (Pd^10^_Cub_), 11 mL of an aqueous solution containing given amounts of PVP (MW ≈ 55 000, 105 mg), K_2_PdCl_4_ (57 mg), NaBr (300 mg) and ascorbic acid (60 mg) were mixed together in water and then heated for 3 h at 80°C. The cleaning of this sample was performed as suggested by Zalineeva et al. (2013, 2015) by the addition of NaOH pellets until having an ~1 M NaOH solution. Under these conditions, the nanoparticles precipitate which were finally washed 3–4 times with ultrapure water and finally stored in a water suspension. Finally, and for the sake of comparison, spherical (~3 nm) Pd nanoparticles (Pd^3^_Sp_) were also prepared using a previously published methodology (Vidal-Iglesias et al., 2012). Very briefly, 0.6 mL of an ice-cold and freshly prepared 0.1 M NaBH_4_ solution was added to a 20 mL aqueous solution containing 2.5 × 10–4 M H_2_PdCl_4_ and 2.5 × 10^−4^ M trisodium citrate prepared in a glass beaker at room temperature. The resulting solution was vigorously stirred for 30 s and subsequently keep unperturbed for the next 30 min. Then, a NaOH pellet was directly added to the solution which results in the destabilization of the nanoparticles which slowly precipitate. Once precipitated, the sample was washed 3–4 times with ultrapure water and finally stored in a water suspension.

TEM experiments were performed with a JEOL JEM-2010 microscope working at 200 kV. Samples for TEM analyses were prepared by depositing a small droplet of the nanoparticle water suspension onto a formvar/carbon-coated copper grid. The sample was then allowed to evaporate in the air at room temperature.

### Electrochemical Characterization

The Pd samples were electrochemically characterized in a conventional three-electrode electrochemical cell at room temperature. An Autolab PGSTAT302N (Metrohm Autolab) system was used to control the electrode potential. Potentials were measured against a reversible hydrogen electrode connected to the cell through a Luggin capillary. A platinum wire was employed as a counter electrode. Working electrodes were prepared by mounting glassy carbon (GC) disks (3 mm diameter, Goodfellow) into Teflon holders. The surface of the electrodes was polished to a mirror finish with 1.0 and 0.3 μm alumina slurries (Buehler). After polishing, the electrodes were sonicated in Milli-Q water (Millipore) in order to remove polishing residues. The catalyst ink (2–5 μL) was pipetted onto the GC electrode surface and the solvent was evaporated at room temperature in an Ar atmosphere.

Pd single crystal electrodes [Pd(111), Pd(100), and Pd(110)], ~2 mm in diameter, were manufactured using the Clavilier method (Clavilier et al., 1980; Hoshi et al., 2000). After the electrodes were flame annealed and cooled down, they were protected by a droplet of ultrapure water and transferred into the electrochemical cell.

The surface cleanness and surface structure of the Pd nanoparticles was evaluated in an Ar-saturated 0.1 M H_2_SO_4_ solution prepared from Millipore Milli-Q water and Merck 96% H_2_SO_4_ deaerated with Ar (99.99%, Air Liquide). For this purpose, the potential was recorded between 0.1 and 0.7 V at 50 mV s^−1^, being the lower potential limited to 0.1 V to avoid hydrogen absorption. Then, to completely clean the surface of the samples, carbon monoxide (CO, 99.997% Air Liquide) was adsorbed onto the Pd catalyst (bubbling CO through the electrolyte at 0.1 V) until the surface was fully CO-covered (verified by cycling the electrode between 0.1 and 0.35 V). Once the surface was checked to be fully blocked, CO was removed from the solution by bubbling Ar (10–12 min for every minute CO was dosed to the solution). Finally, the CO monolayer was electrochemically stripped off from the surface of the Pd particles by sweeping the potential up to 1.0–1.1 V. The subsequent voltammogram corresponding to the CO-free surface was recorded afterwards, both to confirm the absence of CO in the solution and to have the voltammetric profile of the clean nanoparticles. With this latter curve, the active surface area of the Pd nanoparticles was determined with the charge involved in the so-called hydrogen underpotential deposition (UPD) region (between 0.10 and 0.60 V) assuming 212 μC cm^−2^ for the total charge, measured in H_2_SO_4_, after the subtraction of the double-layer charging contribution (Woods, 1976).

Cu UPD experiments were performed in a 0.1 M H_2_SO_4_ + 1 mM CuSO_4_ (Merck, p.a.) + 1 mM NaCl (Fluka, p.a.) solution. The electrode was immersed at a potential of 0.75 V and cycled until a lower potential limit of about 0.2–0.35 V, depending of the nature of each sample, to avoid bulk Cu deposition at the surface of the Pd nanocrystals (Solla-Gullon et al., 2015).

## Results and Discussion

**Figure 1:**
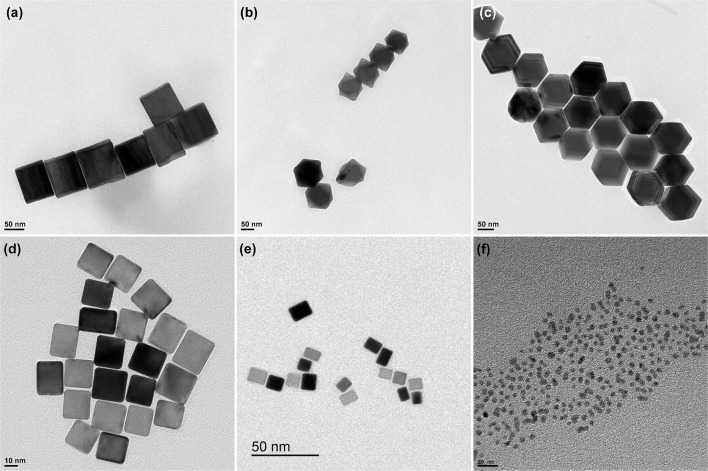
Representative TEM images of the **(a)** Pd_Cub_, **(b)** Pd_Oct_, **(c)** Pd_RD_, **(d)** Pd^20^_Cub_, **(e)** Pd^10^_Cub_, and **(f)** Pd^3^_Sp_ nanoparticles.

Figure 1 displays some representative TEM images of the different Pd nanoparticles synthetized in this work. Figures 1a–c display the shaped Pd nanoparticles prepared using the seed-mediated approach (Niu et al., 2010). These TEM images show well-defined shapes associated with cubic, octahedral and rhombic dodecahedral Pd nanoparticles. It is worth noting that all nanoparticles appear well-separated which is a consequence of the residual presence of PVP or CTAB molecules surrounding the nanoparticles (TEM grids were prepared after centrifugation of the samples and before the incorporation of NaOH). From a statistical analysis of the collected TEM images, we observed a high percentages of cubes (96.0%), octahedra (89.1%) and rhombic dodecahedra (93.3%). The particle size (size edge) of the cubes, octahedra, and rhombic dodecahedra was found to be about 81.2 ± 7.1, 91.7 ± 8.5 51.8 ± 5.2 nm, respectively. The results are in good agreement with those previously reported by Niu et al. (2010). On the other hand, Figures 1d,e show the TEM images of the size controlled Pd nanocubes. The Pd nanocubes prepared in the presence of CTAB display a well-defined cubic shape, a high cubic yield (>90%) and a particle size (size edge) of 21.1 ± 3.0 nm. Similar results were obtained with those prepared in the presence of PVP which also show a well-defined cubic shape, a high cubic yield (>90%) but a smaller particle size (size edge) about 10.1 ± 1.7 nm. It is worth noting that, in these two cases, the preferential cubic shape is related to the interplay of bromide chemisorption on the seeds to promote the formation of {100} facets and an oxidative etching which removes the multiple-twinned seeds (Xiong and Xia, 2007; Xiong et al., 2007; Long et al., 2014). In both cases, the remaining fraction mostly consists of multiplied twinned nanoparticles like decahedra and icosahedra, right bipyramids, nanorods and undefined shapes. (Scardi et al., 2015; Solla-Gullon et al., 2015). Finally, Figure 1f shows the quasi-spherical Pd particles obtained in the presence of citrate which present a particle size (diameter) of about 2.8 ± 0.4 nm.

It is well-accepted that one of the most critical questions to correctly employ these nanomaterials in Electrocatalysis is regarding the cleanness of their surface. However, this important requirement is clearly faced with the need to use capping or surface-stabilizing agents to obtain shaped nanoparticles. Consequently, efficient decontamination protocols to completely remove these capping or surface-stabilizing agents from the surface of the nanoparticles are required (Montiel et al., 2017). Fortunately, as previously stated in the experimental section, the NaOH chemical cleaning (alkaline treatment) has demonstrated to be very efficient for the removal of the capping agents here used (CTAB, PVP and citrate) (Erikson et al., 2011; Vidal-Iglesias et al., 2012; Zalineeva et al., 2013). In addition, as usual, an additional electrochemical cleaning with CO adsorption and its subsequent stripping was also carried out.

**Figure 2:**
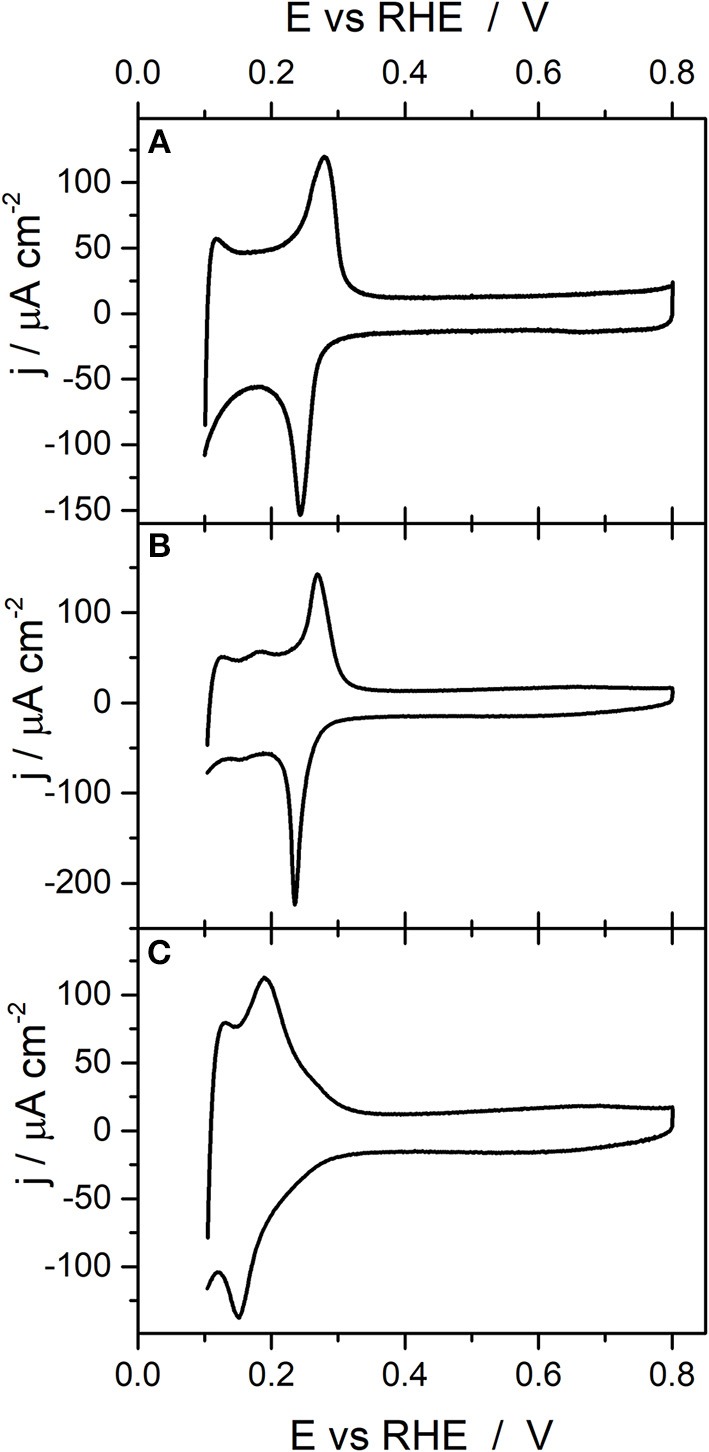
Voltammetric profiles obtained with **(A)** Pd_Cub_, **(B)** Pd_Oct_, and **(C)** Pd_RD_ nanoparticles. Test solution 0.1 M H_2_SO_4_, scan rate 50 mV s^−1^.

Figure 2 shows the voltammetric profiles in the so-called hydrogen/anion adsorption–desorption region obtained with the cubic, octahedral and rhombic dodecahedral Pd nanoparticles. In all cases the lower potential is limited to 0.1 V to avoid hydrogen absorption (Zalineeva et al., 2015). The reported voltammetric results are in good agreement with what is expected from previous single crystal studies (Hoshi et al., 2000, 2002; Wan et al., 2000; Hara et al., 2007). Thus, for the Pd_Cub_ nanoparticles (Figure 2A), anodic and cathodic contributions are observed at about 0.28 and 0.24 V, respectively. These voltammetric features are similar to those observed with Pd(100) and Pd(S)-[*n*(100) × (111)] and Pd(S)-[*n*(100) × (110)] electrodes (Hoshi et al., 2000, 2002; Hara et al., 2007). For the Pd_Oct_ nanoparticles (Figure 2B), the voltammetric profile also shows an anodic feature at 0.27 V and a sharp cathodic one at about 0.235 V, this sharp cathodic contribution being a characteristic feature of Pd(111), Pd(S)-[*n*(111) × (111)] and Pd(S)-[*n*(111) × (100)] electrodes (Hoshi et al., 2000, 2002; Hara et al., 2007). Finally, the voltammetric response of the Pd_RD_ nanoparticles does not show well-defined contributions although two broad peaks were observed at about 0.19 and 0.15 V. A similar poorly defined voltammetric feature was observed for Pd(110) electrodes and also for Pd(S)-[*n*(100) × (111)] and Pd(S)-[*n*(100) × (110)] electrodes with low n values (*n* = 2 and 3) and also for Pd(S)-[*n*(111) × (111)] and Pd(S)-[*n*(111) × (100)] electrodes also with low n values (*n* = 2 and 3) (Hoshi et al., 2000, 2002; Hara et al., 2007). All these findings point out that the cubic, octahedral and rhombic dodecahedral Pd nanoparticles present a preferential {100}, {111}, and {110} surface structure, respectively.

On the other hand, it is also worth mentioning that from the charge involved in this hydrogen/anion adsorption–desorption region, the electroactive surface area of the Pd sample can be easily estimated as previously described in the experimental section. A correct determination of the electroactive surface area is an indispensable point to properly compare the activity of different samples (Fang et al., 2010; Shao et al., 2013b; Moniri et al., 2017; Voiry et al., 2018).

**Figure 3:**
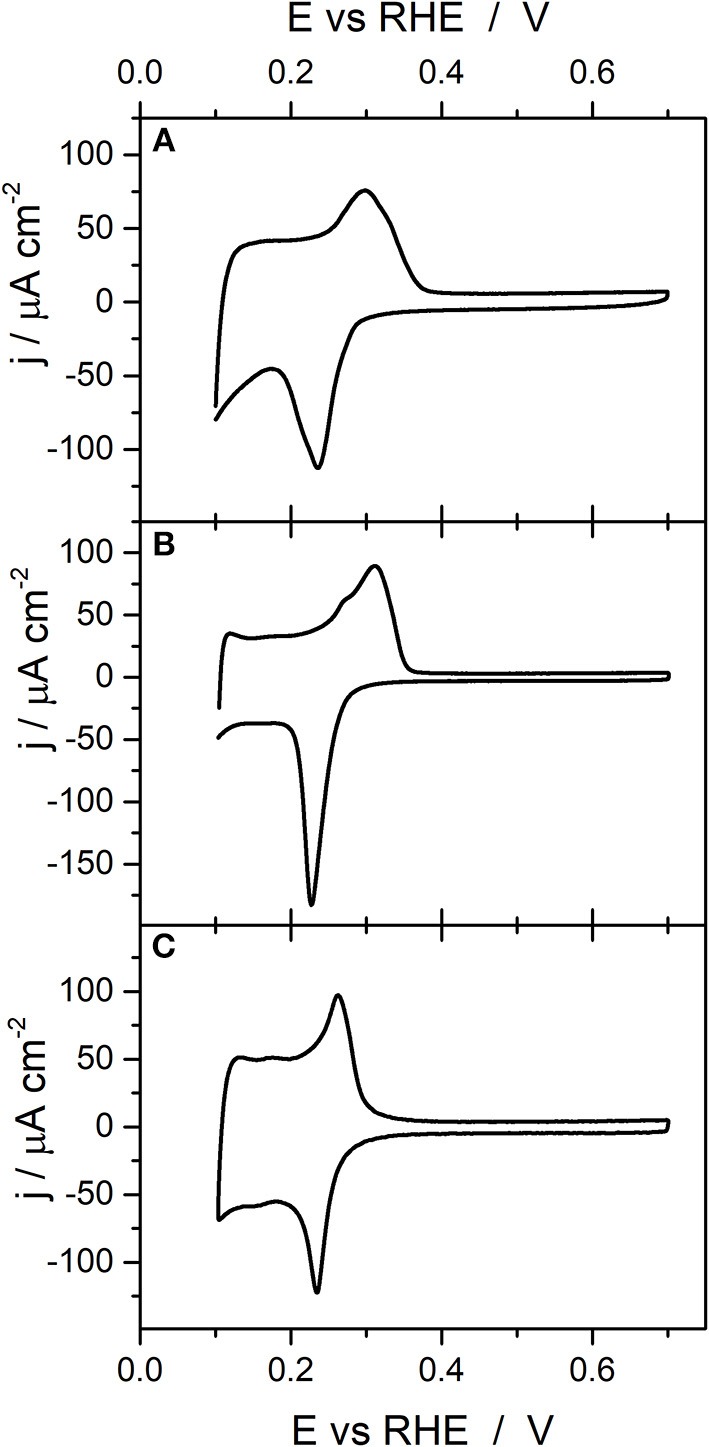
Voltammetric profiles obtained with **(A)** Pd^20^_Cub_, **(B)** Pd^10^_Cub_, and **(C)** Pd^3^_Sp_ nanoparticles. Test solution 0.1 M H_2_SO_4_, scan rate 50 mV s^−1^.

Figure 3 reports the voltammetric profiles obtained with the ~20 nm and ~10 nm cubic Pd nanoparticles as well as that obtained with the quasi-spherical (~3 nm) Pd nanoparticles. The voltammogram obtained with the Pd^20^_Cub_ nanoparticles is similar to that observed in previous contributions with similar samples (Erikson et al., 2011; Vidal-Iglesias et al., 2012; Solla-Gullon et al., 2015) and displays voltammetric features associated with a preferential {100} surface structure. In particular, the voltammogram presents an anodic broad peak centered at about 0.296 V and a cathodic one at about 0.236 V. The presence of broader peaks in comparison with the Pd_Cub_ sample (~80 nm) suggests the existence of a less uniform distribution of {100} domains and/or a higher contribution of other surface sites. A similar trend is also observed with the Pd^10^_Cub_ nanoparticles. However, in this case, the cathodic contribution is clearly sharper than that obtained with the Pd_Cub_ (~80 nm) and Pd^20^_Cub_ nanoparticles, which suggests the presence of a higher contribution of {111} surface domains. Finally, the voltammetric response of the quasi-spherical (~3 nm) Pd nanoparticles is similar to that characteristic of a polyoriented Pd surface (Burke and Nagle, 1999; Vidal-Iglesias et al., 2012).

**Figure 4:**
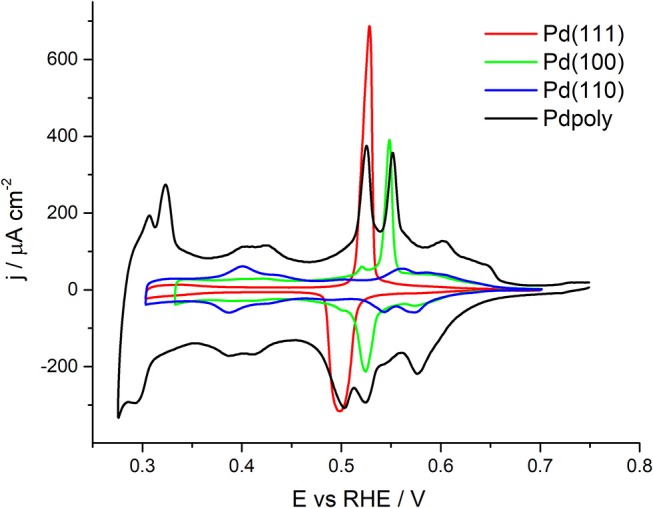
Cu UPD voltammograms obtained with the three Pd single crystal basal planes and with a polyoriented Pd electrode. Test solution 0.1 M H_2_SO_4_ + 1 mM Cu^2+^ + 1 mM NaCl, scan rate 50 mV s^−1^.

From the voltammetric responses shown in Figures 2, 3, it is possible to infer some qualitative information about the surface structure of the different shape and size-controlled Pd nanoparticles. However, it would highly desirable to have some electrochemical surface probe to quantitatively evaluate the surface structure of these nanoparticles. In this sense, Cu UPD on Pd is an interesting process because it has been reported to be sensitive to the surface structure of the Pd electrodes (Chierchie and Mayer, 1988; Cuesta et al., 1999; Herrero et al., 2001; Vidal-Iglesias et al., 2006; Mayet et al., 2019). To evaluate this surface structure sensitivity, Figure 4 shows the Cu UPD profiles obtained in a 0.1 M H_2_SO_4_ + 1 mM CuSO_4_ + 1 mM NaCl solution with the three Pd basal planes (Pd(111), Pd(100), and Pd(110) and with a polyoriented Pd bead. In all cases the lower potential limit is limited exclusively to the Cu UPD region, that is, the bulk Cu deposition and its subsequent stripping is avoided (see Supplementary Figure 1) In addition, the response of the polyoriented Pd surface is multiplied by an arbitrary number to better correlate their different contributions with those obtained with the Pd basal electrodes. The results obtained are in agreement with those previously reported in the literature (Chierchie and Mayer, 1988; Rigano et al., 1990; Cuesta et al., 1999; Okada et al., 2001). For the Pd(111) electrode, well-defined anodic and cathodic peaks at 0.53 and 0.50 V are observed, respectively. The Cu UPD on Pd(110) is much more complex and, in the anodic region, displays wide features at 0.56 and 0.58 V. Also a couple of contributions at 0.40 and 0.43 V are observed related to the Cu UPD second layer deposition on Pd(110). Finally, the Cu UPD response on Pd(100) again shows well-defined anodic and cathodic peaks, in this case at 0.55 and 0.52 V, respectively. However, and due to some surface imperfections/defects, some small contributions at the potentials corresponding to the other basal planes are also visible. Interestingly, the Cu UPD response on the polyoriented Pd electrode is essentially similar to that previously reported by Chierchie and Mayer (1988) and contains multiple contributions which can be well-correlated with the signals previously observed with the Pd single crystal basal planes. Thus, in the positive going sweep, voltammetric features related to {111} (peak at about 0.52 V), {100} (peak at about 0.55 V), and {110} (multiple contributions between 0.40–0.45 and 0.56–0.66 V) surface domains are evident. Also, some additional contributions can be observed between 0.30 and 0.33 V which were previously related to the second Cu monolayer on {100} surface domains (Cuesta et al., 1999; Vidal-Iglesias et al., 2006). Similar Cu UPD voltammetric profiles can be observed with Pd single crystal electrodes prepared by the “forced deposition” method followed by flame annealing of Pd on Pt single crystal electrodes (Vidal-Iglesias et al., 2006, 2007) (see Supplementary Figure 2). Table 2 recompiles the main features [peak position and full width at half maximum (FWHM)] obtained in Figure 4 with the three palladium basal planes.

**Table 2:** Peak potential and full width at half maximum for the main Cu UPD contributions observed in Figure 4 for the three Pd basal plane electrodes.

**Surface**	**Main peak/V**	**FWHM/mV**
Pd(111)	0.529	10
Pd(100)	0.548	7
Pd(110)	0.587 and 0.606	25 and 54

**Figure 5:**
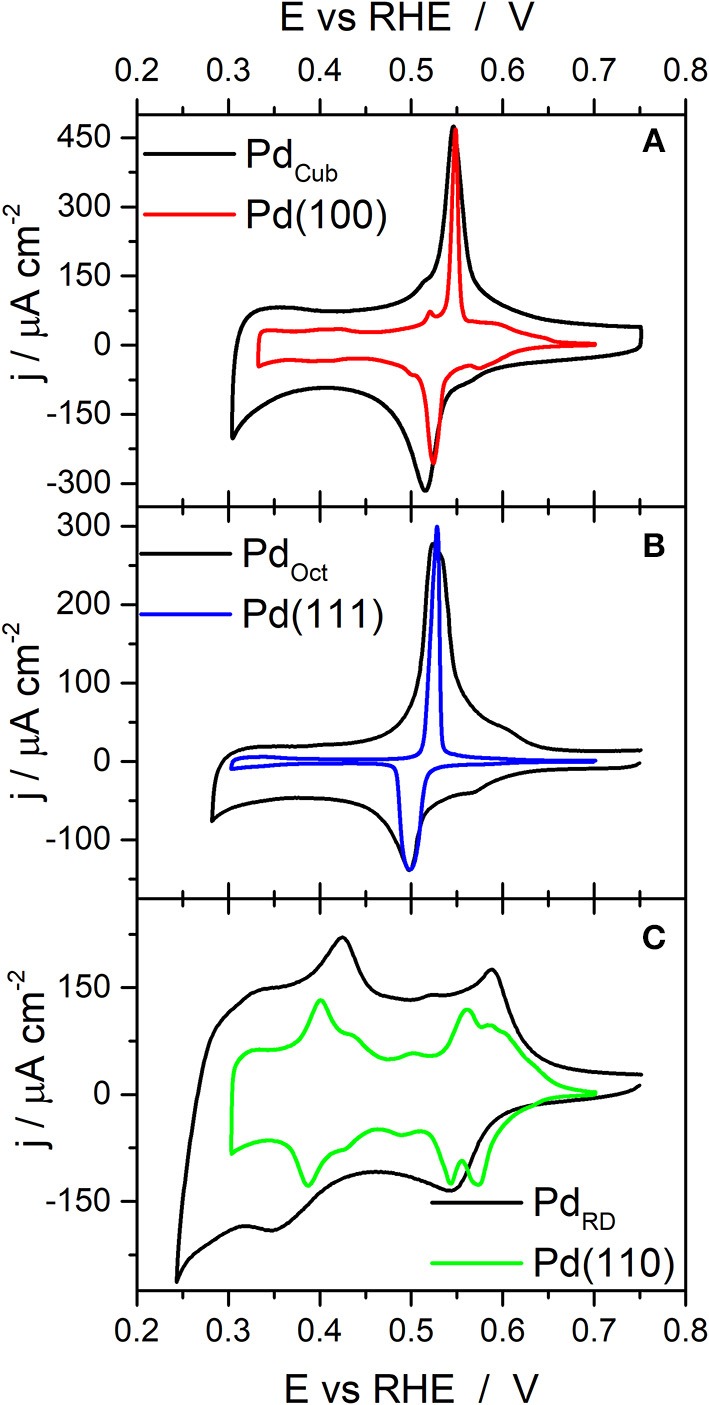
Cu UPD voltammograms obtained with **(A)** Pd_Cub_, **(B)** Pd_Oct_, and **(C)** Pd_RD_ nanoparticles. Test solution 0.1 M H_2_SO_4_ + 1 mM Cu^2+^ + 1 mM NaCl, scan rate 50 mV s^−1^.

The Cu UPD voltammetric profiles obtained with the Pd_Cub_, Pd_Oct_, and Pd_RD_ nanoparticles are reported in Figure 5. Assuming that these shaped Pd nanoparticles will have a preferential {100}, {111}, and {110} surface structure, the Cu UPD response of the corresponding Pd basal planes is also included for the sake of comparison. The results clearly demonstrated that, as expected, the cubic, octahedral and rhombic dodecahedra Pd nanoparticles show a preferential {100}, {111}, and {110} surface structure. However, it is worth noting that the Cu UPD voltammetric features observed with the nanoparticles are broader as a consequence of the presence of domains with different terrace widths. In addition, some minor contributions due to the surface defects including truncations, steps, edges, etc. are also observed. For instance, in case of the Pd_Oct_, some features related to {110} contributions are visible between 0.56 and 0.65 V.

**Figure 6:**
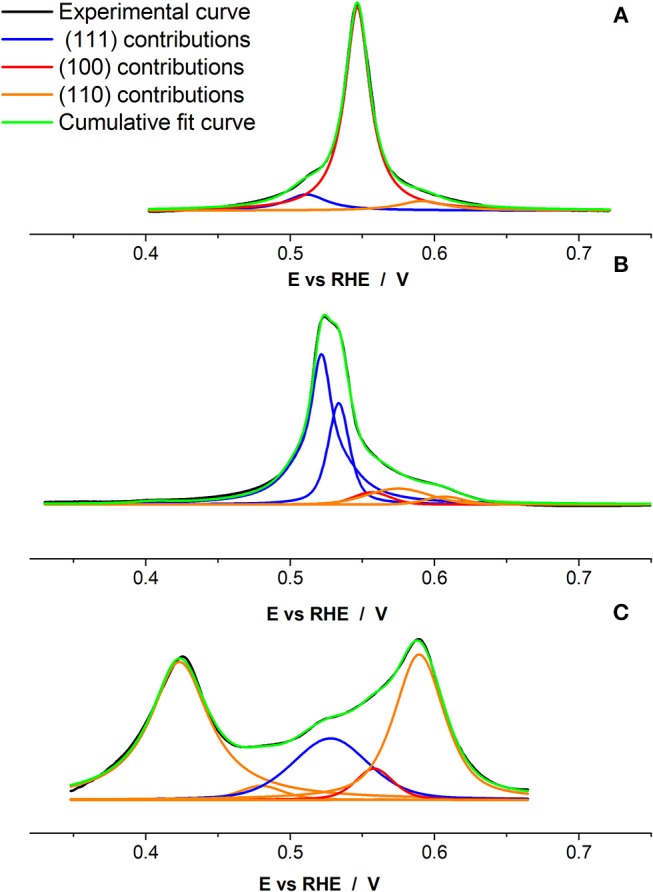
Cu UPD deconvolution with pseudo-Voigt function for the **(A)** Pd_Cub_, **(B)** Pd_Oct_, and **(C)** Pd_RD_ nanoparticles. Test solution 0.1 M H_2_SO_4_ + 1 mM Cu^2+^ + 1 mM NaCl, scan rate 50 mV s^−1^.

Once qualitatively demonstrated that the shaped Pd nanoparticles present a preferential surface structure according with their particle shape, the objective is evaluating if this Cu UPD can also provide quantitative information about the {100}, {111}, and {110} surface domains present at the surface of each type of nanoparticles. With this objective in mind, the Cu UPD voltammetric profiles have been fitted using pseudo-Voigt functions located at the characteristic contributions of the {100}, {110}, and {111} surface domains previously obtained from the Pd single crystal experiments and reported in Figure 4. The percentages of {100}, {110}, and {111} surface sites have been calculated from the area of each corresponding signal in relation to the total area of the Cu UPD process. However, certain questions must be taken into consideration in order to carry out this calculation correctly. Firstly, the contributions between ~ 0.3 and ~0.5 V cannot be used in the analysis because it is accepted they are related to the formation of a second Cu layer on Pd(100) and Pd(110), respectively (Chierchie and Mayer, 1988; Cuesta et al., 1999; Vidal-Iglesias et al., 2006). Secondly, due to the fact that for the three Pd basal planes the area corresponding to Cu UPD process is different, the area of the different contributions between 0.5 and 0.75 V must be also normalized by considering the calculated charges for a complete Cu monolayer on Pd(111), Pd(100), and Pd(110) single crystals (486, 421, and 297 μC cm^−2^, respectively) (Cuesta et al., 1999; Solla-Gullon et al., 2015). Therefore, and using as normalization value the charge for a complete Cu monolayer on a Pd(110) electrode, the contributions related to the {111} and {100} must be divided by 1.64 (486/297) and 1.42 (421/297), respectively. Figure 6 shows the deconvolution of the Cu UPD curves shown in Figure 5. In all cases, the cumulative curve fits well with the experimental one. Table 3 summarizes the main results of this deconvolution process. It is worth noting that in the case of the octahedral Pd nanoparticles (Figure 6B), the main contribution centered at 0.53 V is, in fact, composed of two very close but discernible contributions. This feature which is not observed on Pd(111), may suggest the presence of a bimodal distribution of {111} surface domains.

**Table 3:** Main results of the deconvolution process of the Cu UPD voltammograms on Pd_Cub_, Pd_Oct_, and Pd_RD_ nanoparticles shown in Figure 6.

	**{111} domains**	**{100} domains**	**{110} domains**	**% main shape/TEM**
Pd_Cub_	1 peak	1 peak	1 peak	96
Peak position/V	0.511	0.547	0.591	
FWHM/mV	35	21	39	
% domain	9.0	81.0	10.0	
Pd_Oct_	1 peak and shoulder	1 peak	2 peaks	89
Peak position/V	0.522 and 0.534	0.557	0.575 and 0.607	
FWHM/mV	21 and 18	30	51 and 31	
% domain	54.8 and 22.5	4.5	14.0 and 4.2	
Pd_RD_	1 peak	1 peak	1 peak	93
Peak position/V	0.531	0.558	0.589	
FWHM/mV	62	31	41	
% domain	24.8	5.0	70.2	

The results included in Table 3 are in reasonably good agreement with the statistic obtained from the TEM analysis [cubes (96.0%), octahedra (89.1%), and rhombic dodecahedra (93.3%)]. For the cubic Pd nanoparticles, the deconvolution indicates that these present an 81% of {100} domains and a 9 and 10% of {111} and {110} sites, respectively. The octahedral ones show a 77.3% of {111} domains and a 4.5 and 18.2% of {100} and {110} sites, respectively. Finally, the rhombic dodecahedral Pd nanoparticles display a 70.2% of {110} domains and a 24.8 and 5% of {111} and {100} sites, respectively. As expected, the % of preferential surface sites is lower than the % of preferential shapes. In this sense, it is worth remarking that the surface of the nanoparticles, even nanoparticles with a well-defined particle shape, is not perfect and will also contain a considerable amount of surface defects (steps, edges, corners, kinks) which also including concave and convex domains or rounded and truncated corners. This is also supported by the much wider full width at half maximum (FWHM) of the peaks compared to that obtained for the single crystals.

**Figure 7:**
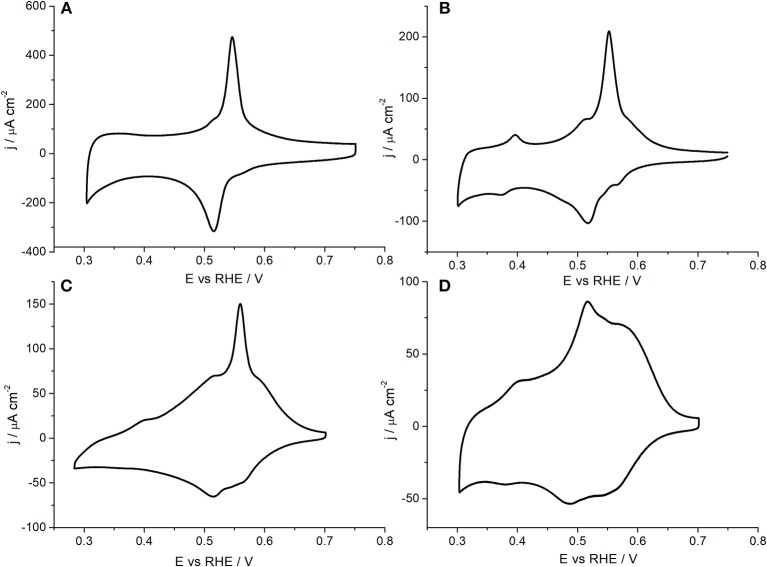
Cu UPD voltammograms obtained with **(A)** Pd_Cub_, **(B)** Pd^20^_Cub_, **(C)** Pd^10^_Cub_, and **(D)** Pd^3^_Sp_ nanoparticles. Test solution 0.1 M H_2_SO_4_ + 1 mM Cu^2+^ + 1 mM NaCl, scan rate 50 mV s^−1^.

**Figure 8:**
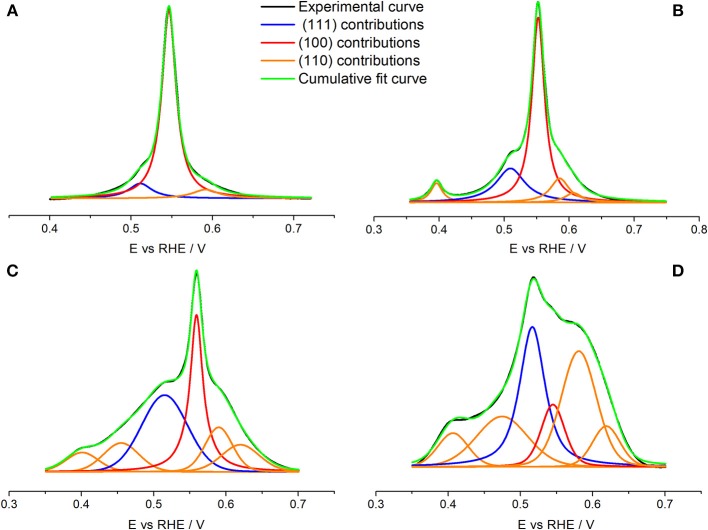
Cu UPD deconvolution with pseudo-Voigt function for **(A)** Pd_Cub_, **(B)** Pd^20^_Cub_, **(C)** Pd^10^_Cub_, and **(D)** Pd^3^_Sp_ nanoparticles. Test solution 0.1 M H_2_SO_4_ + 1 mM Cu^2+^ + 1 mM NaCl, scan rate 50 mV s^−1^.

The effect of particle sizes has been previously studied for many electrochemical reactions (Kinoshita, 1990; Shao et al., 2011a). This type of study is particularly interesting in shaped nanoparticles, for which a decrease in their particle size may produce a significant decrease in their preferential surface structure. For example, cubic particles of different sizes have been used for oxygen reduction, and glucose and formic acid oxidations (Erikson et al., 2011, 2016a; Shao et al., 2011b, 2013a; Vidal-Iglesias et al., 2012; Lüsi et al., 2016; Ye et al., 2016; Vara et al., 2017). Figure 7 shows the Cu UPD voltammetric profiles obtained with the sized controlled cubic Pd nanoparticles (~80, 20, and 10 nm) prepared in this work together with that of the spherical (~3 nm) Pd nanoparticles. The reported results show how the preferential {100} surface structure, associated with the peak at about 0.55 V is, although clearly visible, qualitatively less preferential for decreasing particle sizes. Thus, by decreasing the size, the signals attributed to other surface domains become systematically more perceptible. Finally, for the spherical (~3 nm) Pd nanoparticles, the signal related to the {100} surface site is hardly discernible. To quantitatively evaluate this loss of preferential {100} for decreasing particle size, the Cu UPD responses were deconvoluted as previously discussed in Figure 6. These results are shown in Figure 8 and the main results are summarized in Table 4.

**Table 4:** Main results of the deconvolution process of the Cu UPD voltammograms on (a) Pd_Cub_, (b) Pd^20^_Cub_, (c) Pd^10^_Cub_, and (d) Pd^3^_Sp_ nanoparticles shown in Figure 8.

	**{111} domains**	**{100} domains**	**{110} domain**	**% main shape/TEM**
Pd_Cub_	1 peak	1 peak	1 peak	96
Peak position/V	0.511	0.547	0.591	
FWHM/mV	35	21	39	
% domain	9	81.0	10.0	
Pd^20^_Cub_	1 peak	1 peak	2 peaks	91.9
Peak position/V	0.510	0.552	0.585 and 0.607	
FWHM/mV	53	23	31 and 57	
% domain	21.0	57.4	15.9 and 5.7	
Pd^10^_Cub_	1 peak	1 peak	2 peaks	92.6
Peak position/V	0.515	0.559	0.590 and 0.620	
FWHM/mV	75	22	45 and 62	
% domain	32.2	32.9	19.0 and 15.9	
Pd^3^_Sp_	1 peak	1 peak	2 peaks	100
Peak position/V	0.516	0.545	0.581 and 0.619	
FWHM/mV	42	39	58 and 62	
% domain	30.4	12.3	44.9 and 12.4	

The results obtained show that the % of {100} surface domains decreases from 81% to 57 and then to 33% for decreasing particle sizes from 80 to 20 nm and then to 10 nm, respectively. However, from the TEM statistic, the fraction of cubes was always higher than the 90%. This finding again points out that, for decreasing particle sizes, the contribution of low-coordinated Pd atoms such as edges or corners will represent a larger percentage of the nanoparticles' surface. Obviously, the particular % of surface domains will be strongly affected by the method used for the synthesis, that is, nanoparticles with similar shape and size may have a different % of surface sites as a function of the “quality” of their surface. Finally, the spherical Pd nanoparticles shows a polyoriented surface containing a 57.3% of {110} sites and a 30.4 and 12.3% of {111} and {100} sites, respectively. In summary, the results here reported clearly indicate that Cu UPD is a valuable tool to get insights of the surface structure of different Pd nanoparticles including shape and size-controlled ones.

## Conclusions

In this work, we have demonstrated that Cu UPD is a powerful tool to qualitatively and quantitatively analyse the surface structure of different shape and size controlled Pd nanoparticles. The detailed knowledge of the surface structure of these Pd nanoparticles is particularly important to understand and compare the electrocatalytic activity of these nanomaterials toward relevant surface structure sensitive reactions such as oxygen and CO_2_ reduction or the oxidation of small organic molecules, among many others. The Cu UPD results clearly show that the cubic, octahedral and rhombic dodecahedral Pd nanoparticles prepared using the seed-mediated method present a preferential {100}, {111}, and {110} surface structure, respectively, with percentages of the corresponding domains higher than the 70% in all cases. On the other hand, for the size-controlled Pd nanocubes, the decrease of the particle size produces an evident loss of the {100} quality of the Pd nanocubes despite the fraction of cubes, as deduced from the TEM statistic, remains higher than 90%. This finding again evidences that the electrochemical response is determined by the surface structure but not by the shape or size of the particle.

## Data Availability

The datasets generated for this study are available on request to the corresponding author.

## Author Contributions

EG, FV-I, and JS-G carried out the synthesis, characterization, and electrochemical analysis of the Pd samples. JF and JS-G supervised the work. All authors contributed to write and revise the manuscript.

### Conflict of Interest Statement

The authors declare that the research was conducted in the absence of any commercial or financial relationships that could be construed as a potential conflict of interest.
