# Supplementary material for: Surface Structure Characterization of Shape and Size Controlled Pd Nanoparticles by Cu UPD: A Quantitative Approach
Source: Front Chem. 2019 Jul 31;7:527. doi: 10.3389/fchem.2019.00527 (PMC6684747; doi:10.3389/fchem.2019.00527)
Supplement: Supplementary file 1 [file Data_Sheet_1.docx]

Supplementary Material

**Supplementary Figure 1.** Cu UPD on a polyoriented Pd bead also showing the bulk Cu deposition and stripping. Test solution 0.1 M H_2_SO_4_ + 1mM CuSO_4_ + 1mM NaCl, scan rate 50 mV s^-1^.

**Supplementary Figure 2.** Cu UPD on a polyoriented Pd bead and on Pd single crystal electrodes prepared by the ‘forced deposition’ method followed by flame annealing of Pd on Pt single crystal electrodes [1, 2]. Test solution 0.1 M H_2_SO_4_ + 1mM CuSO_4_ + 1mM NaCl, scan rate 50 mV s^-1^.

**References**.

[1] F.J. Vidal-Iglesias, A. Al Akl, D.J. Watson, G.A. Attard, A new method for the preparation of PtPd alloy single crystal surfaces, Electrochem. Commun. 8 (2006) 1147-1150.

[2] F.J. Vidal-Iglesias, A. Al-Akl, D. Watson, G.A. Attard, Electrochemical characterization of PtPd alloy single crystal surfaces prepared using Pt basal planes as templates, J. Electroanal. Chem. 611 (2007) 117-125.
